# Age-Related Alterations in DTI Metrics in the Human Brain—Consequences for Age Correction

**DOI:** 10.3389/fnagi.2021.682109

**Published:** 2021-06-15

**Authors:** Anna Behler, Jan Kassubek, Hans-Peter Müller

**Affiliations:** Department of Neurology, University of Ulm, Ulm, Germany

**Keywords:** diffusion tensor imaging, age dependence, magnetic resonance imaging, fractional anisotropy, diffusivity

## Abstract

**Background:** Over the life span, the diffusion metrics in brain MRI show different, partly nonlinear changes. These age-dependent changes also seem to exhibit regional differences with respect to the brain anatomy. The age correction of a study cohort's diffusion metrics might thus require consideration of age-related factors.

**Methods:** Diffusion tensor imaging data sets were acquired from 219 healthy participants at ages between 19 and 81 years. Fractional anisotropy (FA), mean diffusivity (MD), and axial and radial diffusivity (AD and RD, respectively) maps were analyzed by a tract of interest-based fiber tracking approach. To describe diffusion metrics as a function of the participant age, linear splines were used to perform curve fitting in 21 specific tract systems covering different functional areas and diffusion directions.

**Results:** In the majority of tracts, an interpolation with a change of alteration rate during adult life described the diffusion properties more accurately than a linear model. Consequently, the diffusion properties remained relatively stable until a decrease (of FA) or increase (of MD, AD, and RD) started at a region-specific time point, whereas a uniform change of diffusion properties was observed only in a few tracts. Single tracts, e.g., located in the cerebellum, remained nearly unaltered throughout the ages between 19 and 81 years.

**Conclusions:** Age corrections of diffusion properties should not be applied to all white matter regions and all age spans in the same way. Therefore, we propose three different approaches for age correction based on fiber tracking techniques, i.e., no correction for areas that do not experience age-related changes and two variants of an age correction depending on the age range of the cohort and the tracts considered.

## Introduction

Diffusion tensor imaging (DTI) is a magnetic resonance imaging (MRI)-based tool to quantitatively determine diffusion properties and assess microstructural properties of white matter (WM) fiber systems by measuring the differences in constraints on water diffusion in different types of tissue (Basser et al., 1994). In this framework, fractional anisotropy (FA) is a measure of the degree of anisotropic water diffusion within a region. The mean diffusivity (MD) describes the magnitude of the mobility of water molecules independent of any tissue directionality, axial diffusivity (AD) refers to the magnitude of diffusion parallel to axonal fiber tracts (FTs), and radial diffusivity (RD) refers to diffusion in the direction perpendicular to the axonal fibers (Le Bihan et al., 2001). Thus, by using DTI, diffusivity in human brain WM can be noninvasively mapped to quantify, first, the directional dependence via FA mapping and, second, the reconstruction of FTs by fiber tracking techniques (Mori et al., 2002). The development of tract-wise analysis of FA values, i.e., reconstruction of tracts of interest (TOIs) and statistical analysis of the FA values along these tracts, allowed for a tract-specific analysis of WM integrity along certain (disease-specific) tracts (Müller et al., 2007).

A growing number of studies have investigated age-related alterations in diffusion properties, both as region of interest (ROI)- and as TOI-based approaches. Consistently, a widespread age-related decline in FA was reported (Salat et al., 2005; Sullivan et al., 2006, 2010; Hsu et al., 2008; Kennedy and Raz, 2009; Zahr et al., 2009; Michielse et al., 2010; Westlye et al., 2010; Kochunov et al., 2012; Lebel et al., 2012; de Groot et al., 2015; Cox et al., 2016). Parallel to the decrease in FA, studies found elevations of MD (Lebel et al., 2012; de Groot et al., 2015; Cox et al., 2016), AD, and RD in tracts during aging (Zahr et al., 2009; Sullivan et al., 2010). The influence of age on diffusion properties outweighs the influence of other demographic factors like gender (Sullivan et al., 2010; Lebel et al., 2012). However, the effect of aging does not have a uniform regional impact: anterior regions were reported to be subject to stronger age-related changes than posterior regions (Salat et al., 2005; Sullivan et al., 2006; Hsu et al., 2008; Cox et al., 2016).

From a methodological point of view, there is a need to consider and correct for the influence of “normal” or physiological aging on the WM microstructure when performing studies at a group level. Usually, the age correction is based on a linear least square regression. The regression coefficient β, i.e., the slope, is used to remove age-related changes with respect to a certain age, e.g., the median or mean age $\overset{-}{x}$. With the following equation, it is possible to calculate the age-corrected value *x*_*adj,i*_ from the measured *y*_*i*_ at the age *x*_*i*_:

(1) $$\begin{array}{l} y_{adj,i}=y_{i}-\left(x_{i}-\bar{x}\right)\cdot \frac{\beta }{years} \end{array}$$

Previous mathematical descriptions of the age-related FA decrease and MD, RD, and AD increase were heterogeneous, given that they range from linear correlations (Salat et al., 2005; Sullivan et al., 2010; Kochunov et al., 2012) over quadratic (Hasan et al., 2009; Kennedy and Raz, 2009; Westlye et al., 2010; Kochunov et al., 2012) and cubic (Hsu et al., 2008; Michielse et al., 2010) descriptions to Poisson curve approaches (Lebel et al., 2012). The results of nonlinear regressions provide valuable information about peak ages and trend changes that cannot solely be represented by linear fits. By use of a linear regression-based age correction, these underlying higher orders of age-related associations would be underestimated and might increase the error brought in by age correction.

Therefore, we investigated a model for age correction of diffusion properties in different cerebral tract systems which is easy to handle and takes effects from higher orders of age dependencies into account. Our tract-based approach uses piecewise linear regression of diffusion properties over the life span in contrast to a single linear regression.

## Methods

**Figure 1:**
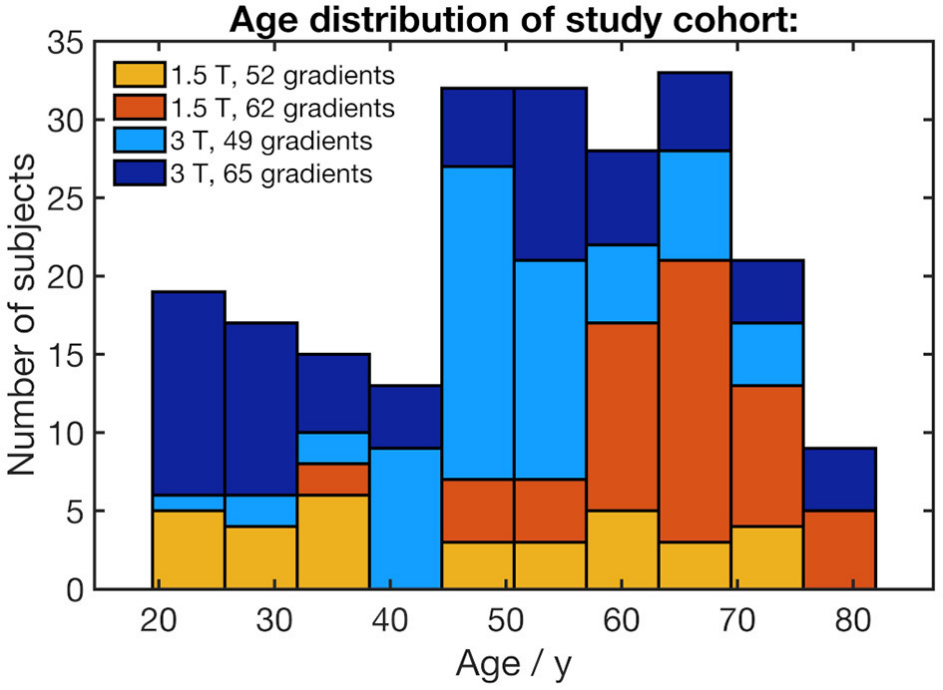
Stacked histogram of the study cohort's age distribution. The age bracket colorization represents the number of subjects who underwent four different scanner protocols.

Two hundred and nineteen healthy adults (103 male/116 female, mean age 51.6 ± 15.9 years, range 19.5–81.9 years) without any history of neurological diseases or any other medical conditions were included. DTI data with artifacts were not included. Gross brain pathology, including vascular brain alterations and microbleedings, could be excluded by conventional MRI including fluid-attenuated inversion recovery and T2-weighted sequences. All subjects gave written informed consent for the study protocol according to institutional guidelines which had been approved by the Ethics Committee of Ulm University, Germany (reference # 19/12 and 279/19). The age distribution for each scanner protocol is shown in Figure 1. The distribution of the subjects' ages with regard to the scanner protocol does not show any clusters that could lead to an unfavorable influence on the scanner correction (see section DTI data processing).

### MRI Data Acquisition

DTI data were acquired by use of four different MRI protocols. On a 1.5-T clinical scanner (Magnetom Symphony, Siemens Medical, Erlangen, Germany), 33 subjects underwent protocol A, and 54 subjects underwent protocol B. Protocol A consisted of 52 gradients including four *b*0 gradient directions (*b* = 1,000 s/mm^2^, voxel size 2.0 mm × 2.0 mm × 2.8 mm, 128 × 128 × 64 matrix, *TE* = 95 ms, *TR* = 8,000 s). Protocol B consisted of 2 × 31 gradient directions including two *b*0 gradient directions (*b* = 1,000 s/mm^2^, voxel size 3.3 mm × 3.3 mm × 3.0 mm, 64 slices, 64 × 64 × 64 matrix, *TE* = 28 ms, *TR* = 3,080 ms). On a 3.0-T MRI scanner (Allegra, Siemens Medical), 64 subjects underwent protocol C consisting of 49 gradients including one *b*0 gradient direction (2.2 mm^3^ isovoxels, 96 × 128 × 52 matrix, *TE* = 85 ms, *TR* = 7,600 ms, *b* = 1,000 s/mm^2^). Finally, 68 subjects underwent protocol D on another 3.0-T MRI scanner (Prisma, Siemens Medical, Erlangen, Germany) with 64 gradient directions and one *b*0 (*b* = 1,000 s/mm^2^, voxel size 2.0 mm × 2.0 mm × 2.0 mm, 58 slices, *TE* = 85 ms, *TR* = 7,600 ms).

### DTI Data Processing

The DTI analysis software Tensor Imaging and Fiber Tracking (TIFT) (Müller et al., 2007) was used for postprocessing following an established analysis protocol (Müller and Kassubek, 2013). DTI data were controlled for eddy current distortions. After resampling to an isotropic 1-mm grid in order to combine the different DTI protocols in a single common resolution frame for further processing steps, spatial nonlinear normalization to the Montreal Neurological Institute (MNI) stereotaxic standard space was iteratively performed on *b*0 and FA template sets for each protocol study group. DTI metrics' maps (FA, AD, MD, and RD) of each subject's data set were smoothed with a Gaussian filter of 8-mm full width at half maximum. For each of protocols A–D, averaged DTI data sets were calculated separately by arithmetic averaging of the MNI normalized data. These averaged data sets were used to calculate difference maps between protocol group D and any other protocol group due to protocol variability, i.e., differences in voxel size, TE and number of gradients, and scanner-specific variability. Differences were then regressed out by 3-D linear correction matrices (Müller et al., 2016). The first-order linear 3-D corrections were then applied to the FA, MD, AD, and RD maps of protocol groups A, B, and C. The use of two different scanners and again two different protocols on each of them and subsequent homogenization leads to a broad applicability of the results to a wide array of applications.

For voxel-wise correlation, the FA values from all subjects were correlated voxel-wise by Pearson correlation to age. Results were corrected for multiple comparisons by a false density rate (FDR) algorithm (Genovese et al., 2002).

### Fiber Tracking

**Figure 2:**
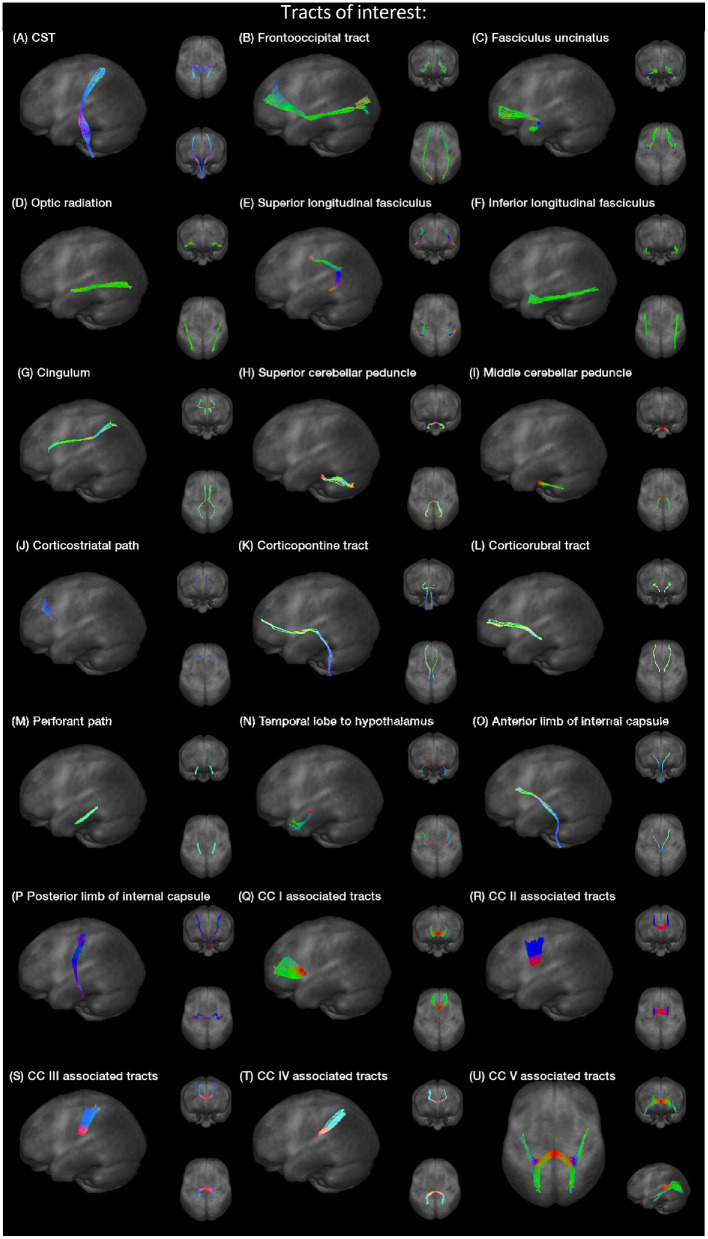
**(A–U)** Projectional views of TOIs (CC, corpus callosum).

Specific tracts were identified by using a seed-based approach based on the averaged protocol D data set, since most subjects underwent protocol D. The modified deterministic streamline tracking approach (Mori et al., 2002; Müller et al., 2009) used an eigenvector scalar product threshold of 0.9. Only voxels with an FA value above a certain threshold, ranging from 0.05 to 0.15 (Table 1), were considered. ROIs with a radius of between 6 and 10 mm were defined for the seed regions. All FTs originating in the seed ROI, or multiple ROIs for extended seed regions (e.g., callosal areas), define the corresponding TOIs (Figure 2). Diffusion metrics were calculated by arithmetic averaging of the bihemispheric data.

**Table 1:** Montreal Neurological Institute (MNI) coordinates and radius for seed ROIs and the used FA threshold for the respective fiber tracking (CC, corpus callosum; CST, corticospinal tract; SLF, superior longitudinal fasciculus; ILF, inferior longitudinal fasciculus; SCP, superior cerebellar peduncle; MCP, middle cerebellar peduncle; AIC, anterior limb of internal capsule; PIC, posterior limb of internal capsule).

**Tract system**	**Seed, *x*/*y*/*z*, mm**	**FA threshold**	**ROI radius, mm**
CST	±26/−22/18	0.15	10
Fronto-occipital tract	±27/5/1	0.10	10
Fasciculus uncinatus	±36/−3/−13	0.15	10
Optic radiation	±42/−52/1	0.15	10
SLF	±36/−44/29	0.10	10
ILF	±45/−32/−16	0.10	10
Cingulum	±7/11/25	0.10	10
SCP	0/−29/−25	0.15	10
MCP	0/−14/−34	0.15	10
Corticostriatal tract	±23/26/20	0.15	10
Corticopontine tract	±8/−24/−11	0.10	8
Corticorubral tract	±6/−32/−13	0.10	8
Perforant path	±25/−17/−24	0.15	10
Temporal lobe to hypothalamus	±18/−6/−8	0.10	6
AIC	±20/2/15	0.10	8
PIC	±22/−12/10	0.10	8
CC I-associated tracts	0/12/4; 0/17/8; 0/16/7	0.15	10
CC II-associated tracts	0/4/18; 0/−1/21; 0/−6/23	0.15	10
CC III-associated tracts	0/−17/23; 0/−21/23	0.15	10
CC IV-associated tracts	−1/−27/21	0.10	10
CC V-associated tracts	0/−34/17; 0/−36/11; 0/−39/15	0.15	10

Fiber tracking was used to delineate the following tracts, the corticospinal tract (CST), the fronto-occipital tract, the fasciculus uncinatus, the optic radiation, superior and inferior longitudinal fasciculus (SLF and ILF), the cingulum bundle, superior and middle cerebellar peduncle (SCP and MCP), the corticostriatal path, the corticopontine tract, the corticorubral tract, the perforant path, the path from the temporal lobe to hypothalamus, the anterior and posterior limbs of the internal capsule (AIC and PIC) according to *a priori* information on tract location (Nieuwenhuys et al., 2008; Mori et al., 2011), and finally the tracts associated to corpus callosum (CC) areas I–V (Hofer and Frahm, 2006). Projectional views of all investigated tract systems are shown in Figure 2.

### Curve Fitting

Since the knowledge from previous studies (Salat et al., 2005; Sullivan et al., 2010; Lebel et al., 2012; Cox et al., 2016) should be incorporated into the modeling of the age dependence of the diffusion parameters, we performed shape prescriptive modeling using the “SLM” (Shape Language Modeling) MATLAB^®^ package (D'Errico, 2017). This model uses least squares splines with simple constraints for a curve fitting. Possible constraints are based on monotonicity and curvature or value constraints, i.e., some maximum or minimum value or a fixed point the fitted curve must pass through. The position of the knots which are the bounds between the segments of the spline can be adjusted to the needs of the modeling, and their number and position can be predefined.

Diffusion metrics were fitted monotonously for decrease (for FA) or increase (for MD, RD, and AD) in accordance with the findings of previous studies (Salat et al., 2005; Sullivan et al., 2010; Lebel et al., 2012; Cox et al., 2016). The degree of the spline was set to 1, i.e., linear regression for each spline segment. To model a change of the alteration rate during aging, the number of knots was specified to 3: one knot at each end of the age range and one in between leading to two linear segments. The position of the knot in between *x*_*knot*_ was not specified in more detail and can thus be freely adjusted by modeling. For a single linear regression over the whole age span, the number of knots was set to 2, i.e., one knot at minimum and maximum ages, and linear splines was used. Monotonicity was set as decreasing for FA and increasing for MD, RD, and AD. Adjusted coefficients of determination $R_{adj}^{2}$ as a goodness of fit for both fitting procedures were calculated.

### Bootstrapping

To estimate the accuracy of the knot position for two linear segments, the standard deviation (SD) was determined *a posteriori* using bootstrapping of the residuals. Therefore, the following steps were performed:

1. The residuals ${\hat{\varepsilon }}_{i}$, i.e., the difference between the fitted value ŷ_*i*_ and the measured value *y*_*i*_ (which corresponds to age *x*_*i*_) were calculated: ${\hat{\varepsilon }}_{i}=y_{i}-{ŷ}_{i}$.
2. For each *x*_*i*_, a synthetic response variable $y_{i}^{*}$ was created by adding a randomly resampled residual ${\hat{\varepsilon }}_{j}$ to the fitted value ŷ_*i*_: $y_{i}^{*}={ŷ}_{i}$ $+{\hat{\varepsilon }}_{i}$ with *j* ∈ {1, 2, …, 219}.
3. This new synthetic data set ($x_{i},y_{i}^{*}$) was fitted with previous parameters, and the knot positions were determined.
4. Steps 2 and 3 were repeated.

After 1,000 bootstrapping iterations, the SD of the knot positions of the synthetic data were calculated.

## Results

### Voxel-Wise Pearson Correlation

**Figure 3:**
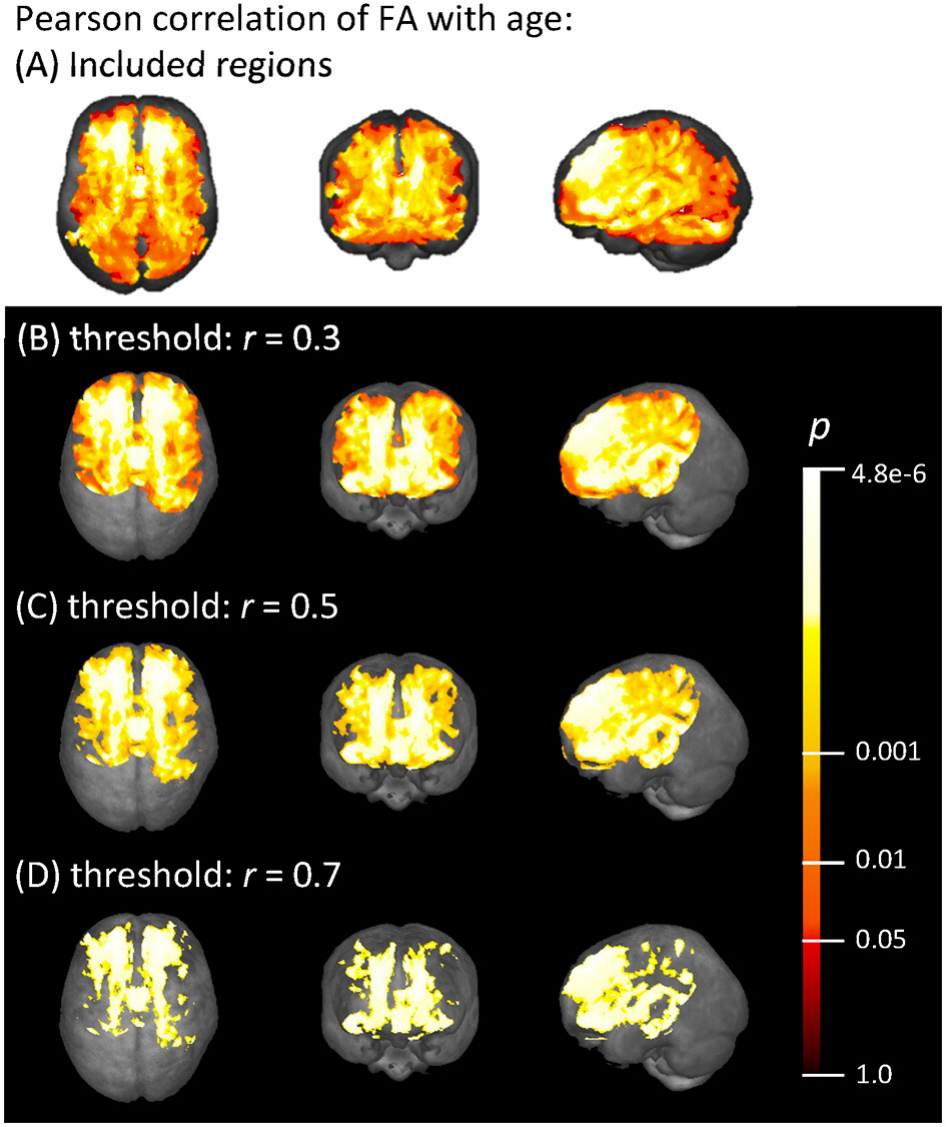
**(A)** Voxels with an FA > 0.2 that were included in the correlation analysis, and **(B–D)** voxel-wise Pearson correlation of FA with age (range 19.5–81.9 years) shown in axial, coronal, and sagittal projectional views, respectively. Correlation coefficient *r* is given above a threshold of **(B)** *r* = 0.3, **(C)** *r* = 0.5, and **(D)** *r* = 0.7, corresponding to increasing strength of the correlation. The color bar represents the *p*-value.

Voxel-wise Pearson correlation of all subjects' FA maps with their individual ages showed a medium effect with *r* > 0.3 in widespread frontal, parietal, temporal, and callosal areas, as demonstrated in Figure 3. The strongest correlation effects (*r* > 0.7) were located in the frontal lobe and in anterior callosal regions. The effect of age on the FA was less pronounced in parietal and temporal areas, since the correlation coefficient in these areas remained below 0.7. There was no significant correlation effect between FA and age in occipital areas and the cerebellum.

### FA

**Figure 4:**
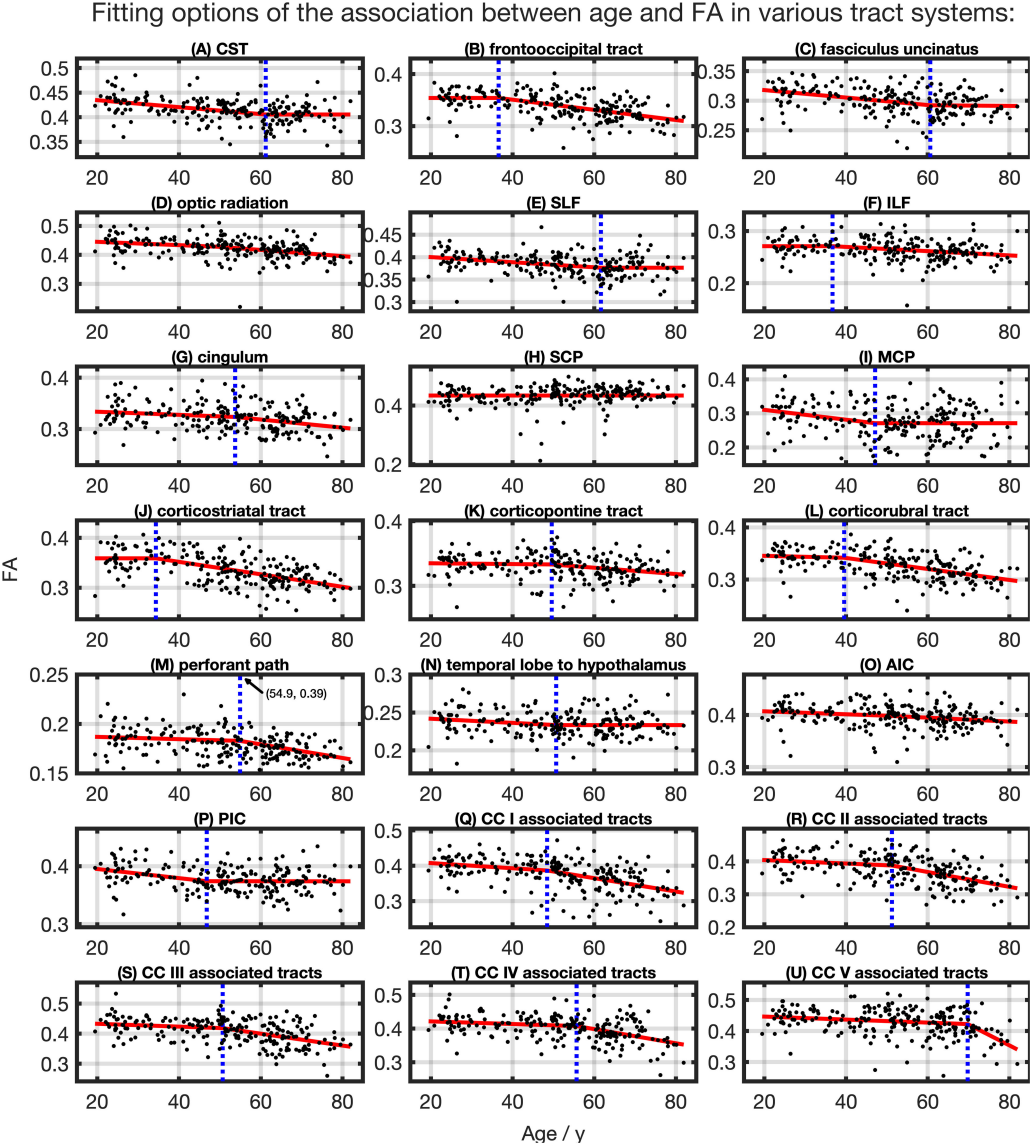
Scatter plots (black) and regression line (red) of the association between age and FA in various tract systems. The fit shown in each case is the one with the highest adjusted *R*^2^. **(D,H,O)** One linear regression across age span. **(A–C,E–G,I–N,P–U)** In all other tract systems, data were fitted with linear splines. The position of the knot between two linear curve segments is given as a blue dashed line (CC, corpus callosum; CST, corticospinal tract; SLF, superior longitudinal fasciculus; ILF, inferior longitudinal fasciculus; SCP, superior cerebellar peduncle; MCP, middle cerebellar peduncle; AIC, anterior limb of internal capsule; PIC, posterior limb of internal capsule).

The relationship between FA and age, shown in Figure 4, was best described by linear spline interpolation in most TOIs, i.e., with two linear curve segments and a knot position in between. The $R_{adj}^{2}$ of the splines was larger than for linear regression in most TOIs over the complete adult life span (Table 2). For the optic radiation, AIC, and SCP (Figures 4D,H,O), the association of age and FA fitted by one linear regression across the whole age range led to a higher value of $R_{adj}^{2}$. The slope in the regression of SCP was negligible (Figure 4H) so that FA could be considered as a constant during adulthood. The knot positions where the change of alteration rate occurs in the linear splines ranged from 34.3 to 69.8 years, and the estimated SDs of these knot positions were around 8.3–10.3 years (Table 3). The change of the alteration rate followed two different patterns: no or just a slight FA decrease in the first curve segment of the splines followed by a more intense decrease in the second curve segment of the interpolation for the fronto-occipital tract; the ILF; the cingulum; the corticostriatal, corticopontine, and corticorubral tracts; the perforant path; all CC-associated tracts or vice versa in the CST; the fasciculus uncinatus; the SLF; the MCP; the path from the temporal lobe to hypothalamus; and the PIC. The decline of FA throughout the life span was not the same for all regions, and the maximum absolute decrease occurred in tracts located in prefrontal areas.

**Table 2:** Adjusted coefficients of determination $R_{adj}^{2}$ of shape prescriptive fits of fractional anisotropy (FA), mean diffusivity (MD), axial diffusivity (AD), and radial diffusivity (RD) over adulthood with two and three knots.

	**$R_{adj}^{2}$ for fit with 3 knots**				**R$R_{adj}^{2}$ for fit with 2 knots**			
	**FA**	**MD**	**AD**	**RD**	**FA**	**MD**	**AD**	**RD**
CST	**0.145**	**0.131**	**0.143**	**0.134**	0.127	0.111	0.096	0.126
Fronto-occipital tract	**0.273**	**0.210**	**0.185**	**0.219**	0.257	0.179	0.145	0.194
Fasciculus uncinatus	**0.132**	**0.226**	**0.243**	**0.213**	0.129	0.192	0.180	0.190
Optic radiation	0.132	**0.230**	**0.216**	**0.229**	**0.132**	0.198	0.181	0.199
SLF	**0.085**	**0.178**	**0.162**	**0.179**	0.074	0.173	0.150	0.177
ILF	**0.057**	**0.386**	**0.379**	**0.384**	0.056	0.335	0.320	0.336
Cingulum	0.091	**0.084**	**0.090**	**0.089**	0.089	0.067	0.070	0.075
SCP	−0.009	**−0.002**	**0.009**	−0.006	**−0.005**	−0.004	0.000	**−0.004**
MCP	**0.048**	**0.010**	**0.008**	**0.013**	0.029	0.004	0.001	0.007
Corticostriatal path	**0.302**	**0.152**	**0.058**	**0.193**	0.287	0.138	0.037	0.186
Corticopontine tract	**0.053**	**0.319**	**0.336**	**0.299**	0.048	0.211	0.216	0.200
Corticorubral tract	**0.269**	**0.198**	**0.180**	**0.208**	0.260	0.166	0.136	0.182
Perforant path	**0.074**	**0.182**	**0.163**	**0.194**	0.064	0.141	0.128	0.153
Temporal lobe to hypothalamus	**0.018**	**0.182**	**0.195**	**0.172**	0.016	0.159	0.163	0.155
AIC	0.046	**0.209**	**0.239**	**0.206**	**0.049**	0.135	0.143	0.149
PIC	**0.080**	**0.186**	**0.182**	**0.186**	0.061	0.166	0.134	0.175
CC I-associated tracts	**0.251**	**0.103**	**0.079**	**0.114**	0.242	0.068	0.036	0.083
CC II-associated tracts	**0.258**	**0.101**	**0.102**	**0.107**	0.231	0.082	0.078	0.087
CC III-associated tracts	**0.221**	**0.123**	**0.124**	**0.125**	0.199	0.084	0.077	0.091
CC IV-associated tracts	**0.195**	**0.133**	**0.148**	**0.129**	0.165	0.088	0.093	0.089
CC V-associated tracts	**0.138**	**0.171**	**0.178**	**0.174**	0.084	0.091	0.111	0.098

*The larger value in the comparison of both fit methods is marked in bold type (CC, corpus callosum; CST, corticospinal tract; SLF, superior longitudinal fasciculus; ILF, inferior longitudinal fasciculus; SCP, superior cerebellar peduncle; MCP, middle cerebellar peduncle; AIC, anterior limb of internal capsule; PIC, posterior limb of internal capsule)*.

**Table 3:** Knot positions of segmented linear regression and standard deviations (SDs) for fractional anisotropy (FA), mean diffusivity (MD), axial diffusivity (AD), and radial diffusivity (RD) in all investigated tract systems.

	**FA**		**MD**		**AD**		**RD**	
	***x_***knot***_*/*y***	**SD of *x_***knot***_*/*y***	***x_***knot***_*/*y***	**SD of *x_***knot***_*/*y***	***x_***knot***_*/*y***	**SD of *x_***knot***_*/*y***	***x_***knot***_*/*y***	**SD of *x_***knot***_*/*y***
CST	61.2	8.4	51.6	8.9	53.8	9.4	51.2	9.8
Fronto-occipital tract	36.6	8.3	58.3	9.5	58.3	9.0	68.0	10.0
Fasciculus uncinatus	60.6	9.3	50.9	8.7	51.0	9.1	50.7	8.9
Optic radiation	–	–	51.0	8.8	52.8	8.6	50.8	8.6
SLF	61.7	8.5	51.0	9.2	51.2	9.0	50.7	7.9
ILF	36.7	8.8	50.7	8.2	51.0	8.8	48.1	8.4
Cingulum	53.7	8.8	58.2	9.9	68.0	10.9	57.7	9.7
SCP	–	–	55.0	9.0	50.7	9.0	–	–
MCP	47.1	9.4	43.8	9.0	44.4	8.1	43.6	9.2
Corticostriatal path	34.3	8.9	53.4	9.0	53.6	8.8	51.2	8.9
Corticopontine tract	49.6	8.7	50.3	7.7	50.7	8.1	50.3	8.1
Corticorubral tract	39.5	9.3	58.6	9.9	58.0	9.0	58.7	9.4
Perforant path	54.9	8.9	50.9	8.3	50.7	8.2	51.0	8.1
Temporal lobe to hypothalamus	50.7	8.5	50.7	8.8	50.6	8.7	50.7	8.8
AIC	–	–	50.7	8.4	51.3	8.3	50.3	8.7
PIC	46.8	9.4	69.5	9.9	58.1	9.7	70.8	9.8
CC I-associated tracts	48.5	8.8	69.6	10.6	69.6	10.7	69.5	10.6
CC II-associated tracts	51.2	8.7	65.6	10.5	62.2	9.5	58.7	8.6
CC III-associated tracts	50.7	8.4	58.7	7.7	58.8	8.5	58.7	7.6
CC IV-associated tracts	55.7	9.3	60.9	8.5	69.0	11.5	60.9	8.4
CC V-associated tracts	69.8	10.3	69.0	11.6	68.0	10.4	69.1	11.3

*If no knot position is specified, the adjusted R^2^ of the unsegmented regression was higher (CC, corpus callosum; CST, corticospinal tract; SLF, superior longitudinal fasciculus; ILF, inferior longitudinal fasciculus; SCP, superior cerebellar peduncle; MCP, middle cerebellar peduncle; AIC, anterior limb of internal capsule; PIC, posterior limb of internal capsule)*.

### MD

**Figure 5:**
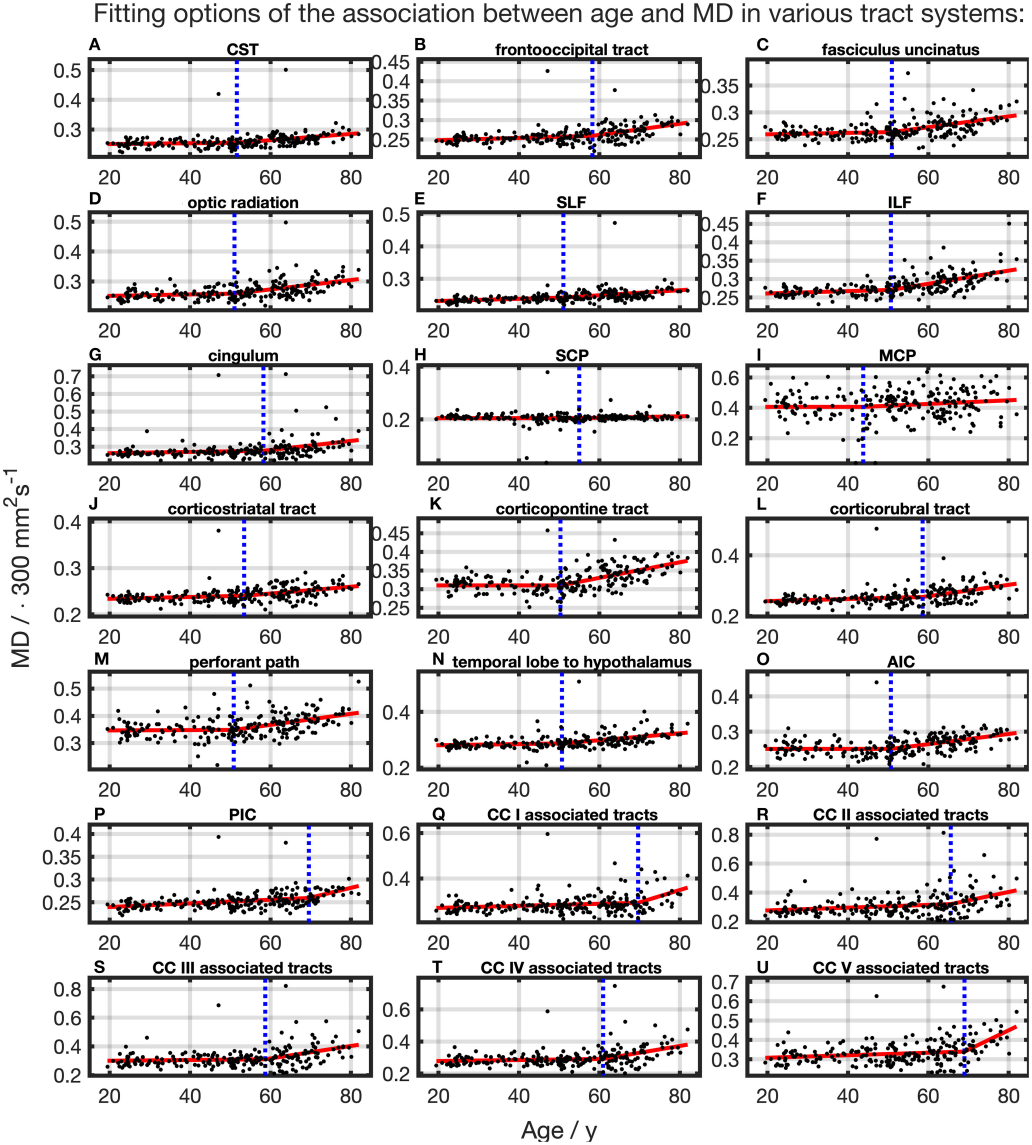
Scatter plots (black) and regression line (red) of the association between age and MD in various tract systems. **(A–U)** In all tract systems, data were fitted with linear splines. The position of the knot between two linear curve segments is given as a blue dashed line (CC, corpus callosum; CST, corticospinal tract; SLF, superior longitudinal fasciculus; ILF, inferior longitudinal fasciculus; SCP, superior cerebellar peduncle; MCP, middle cerebellar peduncle; AIC, anterior limb of internal capsule; PIC, posterior limb of internal capsule).

For all TOIs, curve fitting with two linear curve segments was more accurate than one linear regression curve over complete adulthood due to a higher $R_{adj}^{2}$ (Table 2 and Figure 5). As for the FA, the slope of linear regression in SCP is nearly 0; i.e., MD was not affected here by time. In linear spline interpolations, the knot positions ranged from 43.8 to 69.6 years, and the estimated SDs of these knot positions were around 7.7–11.6 years (Table 3). In contrast to the results in FA, the change of tendency was subject to the same pattern: no or just a slight MD increase in the first segment followed by a more intense increase in the second segment.

### AD

**Figure 6:**
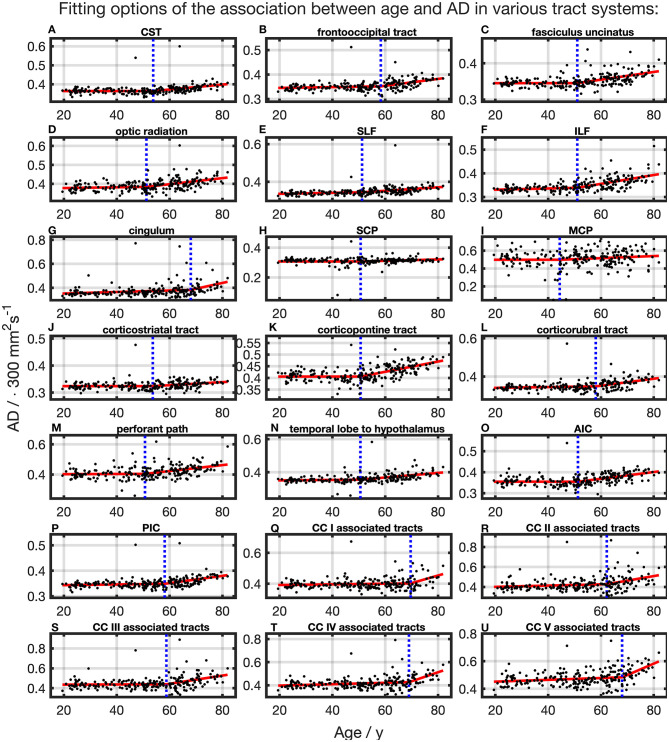
Scatter plots (black) and regression line (red) of the association between age and AD in various tract systems. **(A–U)** All plots are fitted with a segmented linear regression due to a higher adjusted *R*^2^ in comparison to a linear regression fit over the whole age span. The position of the knot between two linear curve segments is given as a blue dashed line (CC, corpus callosum; CST, corticospinal tract; SLF, superior longitudinal fasciculus; ILF, inferior longitudinal fasciculus; SCP, superior cerebellar peduncle; MCP, middle cerebellar peduncle; AIC, anterior limb of internal capsule; PIC, posterior limb of internal capsule).

The association of AD and age, described by fitting with two linear curve segments, had the highest $R_{adj}^{2}$ (Table 2 and Figure 6). Knot positions ranged from 44.4 to 69.6 years (SD range: 8.1 to 11.5 years) as shown in Table 3. The alignment of the two linear curve segments followed the same pattern as MD: no or just a slight increase in the first segment followed by a more intense increase in the second segment. The changes in the SCP were again so small that AD could be regarded as constant here.

### RD

**Figure 7:**
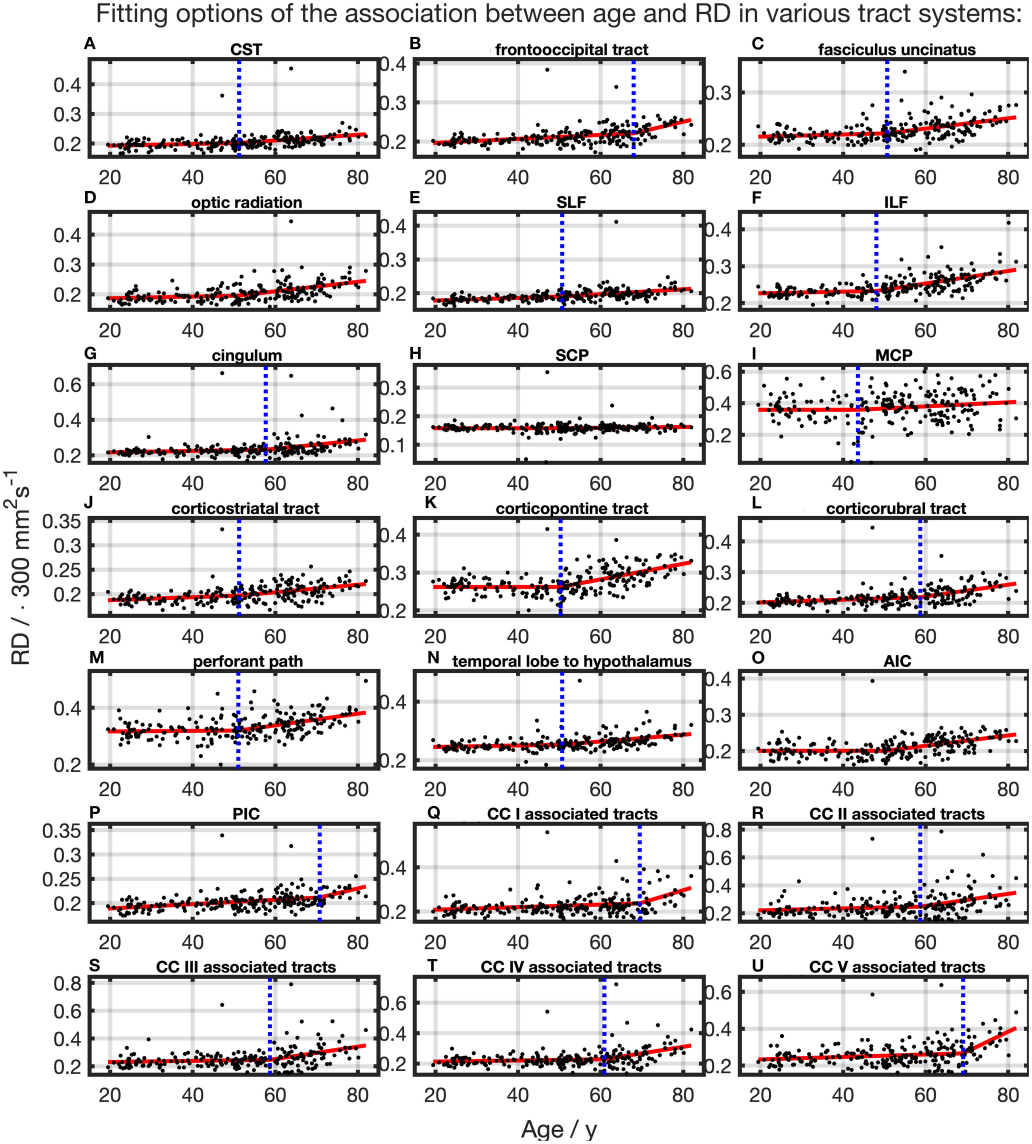
Scatter plots (black) and regression line (red) of the association between age and radial anisotropy (RD) in various tract systems. The fit shown in each case is the one with the highest adjusted *R*^2^. **(H)** One linear regression across age span. **(A–G,I–U)** In all other tract systems: segmented linear regression. The position of the knot between two linear curve segments is given as a blue dashed line (CC, corpus callosum; CST, corticospinal tract; SLF, superior longitudinal fasciculus; ILF, inferior longitudinal fasciculus; SCP, superior cerebellar peduncle; MCP, middle cerebellar peduncle; AIC, anterior limb of internal capsule; PIC, posterior limb of internal capsule).

For the majority of investigated tracts, spline interpolation with two linear curve segments revealed a higher $R_{adj}^{2}$ in comparison to a “classic” linear regression of RD, which had a higher $R_{adj}^{2}$ in SCP (Table 2 and Figure 7). In line with the characteristics of the other diffusion metrics in the SCP, RD was not affected by time. In the fits using linear splines, the knot positions ranged from 43.6 to 70.8 years, and the estimated SDs of these knot positions were around 7.9–11.3 years.

### Consequences for Age Correction

Obviously, the absolute FA, MD, RD, and AD values may differ between tracts; however, this fact does not basically affect the fitting procedure.

Regardless of the fitting method with the smallest adjusted *R*^2^, all diffusion metrics in the SCP and MCP showed only very little change as a function of age. Therefore, age correction would not be applicable for these tracts.

In two tracts, i.e., optic radiation and AIC, a fit of the FA with linear splines resulted in a smaller adjusted *R*^2^ than a linear regression over the entire age range. Therefore, an adjustment of the age correction would not be necessary, and equation (1) should be applied.

Besides these handful of exceptions, an age correction based on a linear regression over the entire age range would not be appropriate to all other tracts in which diffusion metrics showed a change of the alteration rate throughout the life span, i.e., a more accurate fit with linear splines. An adapted version of equation (1) can be used to correct the clearly age-affected values, i.e., the interval [*x*_*knot*_, *x*_*max*_], of the second curve segment for linear splines:

(2) $$\begin{array}{l} y_{adj,i}=y_{i}-\left(x_{i}-x_{knot}\right)\cdot \frac{\beta_{2}}{years} \end{array}$$

## Discussion

In this study, the underlying diffusion metrics for age correction of diffusion properties in different cerebral tract systems was studied in a large database of healthy subjects. With respect to the spatial distribution of age-related effects, our results of voxel-wise age correlation of the diffusion parameters and of the TOI analysis showed a differential pattern in different tracts, in agreement with previous publications (Westlye et al., 2010; de Groot et al., 2015). Frontal and callosal areas are most affected by normal aging. The tract systems studied in the cerebellum, SCP and MCP, show hardly any changes over the age range, consistent with the results of the voxel-wise correlation analysis where no correlations were observed in the cerebellum. We suggest that, due to the spatial differences of age-related changes, tracts to be studied should be corrected individually with respect to their respective age association.

Consistently for all diffusion properties, we found a predominant pattern of age-related alterations. From young adulthood, the characteristics remain relatively stable. The beginning of the deviation from the constant trajectory is mathematically represented by *x*_*knot*_. This finding is in line with a three-phase age-related change of FA, MD, and RD across the life span, as proposed by Westlye et al. (2010). In the first phase, FA increases and MD and RD decrease, followed by a phase of relative stability in diffusion properties until the third phase when FA decreases and MD and RD increase. For age corrections in adult study cohorts, the transition point of time from the second to the third phases apparently takes an essential role. With the help of a segmented linear regression, we were able to map this transition in time for different tract systems. Only in six investigated tract systems (CST, fascicles uncinatus, SLF, MCP, temporal lobe to hypothalamus, and PIC) did the interpolation of FA not follow this described three-phase sequence. During aging, the FA in these tracts initially decreases and then remains stable in advanced age. The fact that this solution is mathematically the most favorable might suggest the existence of a Poisson curve as an age association over the entire life span as proposed by Lebel et al. (2012).

The so-called “brain age” of a subject, estimated by structural properties, often differs from the actual chronological age (Franke et al., 2010; Cole et al., 2017; Smith et al., 2019). Reasons are hard to determine and might be due to the individual lifestyle (Franke et al., 2014). Evaluating the age-related changes in our sample provide insight into the regional magnitude of the alteration process at the group level underlying normal aging regardless of individual influences.

**Figure 8:**
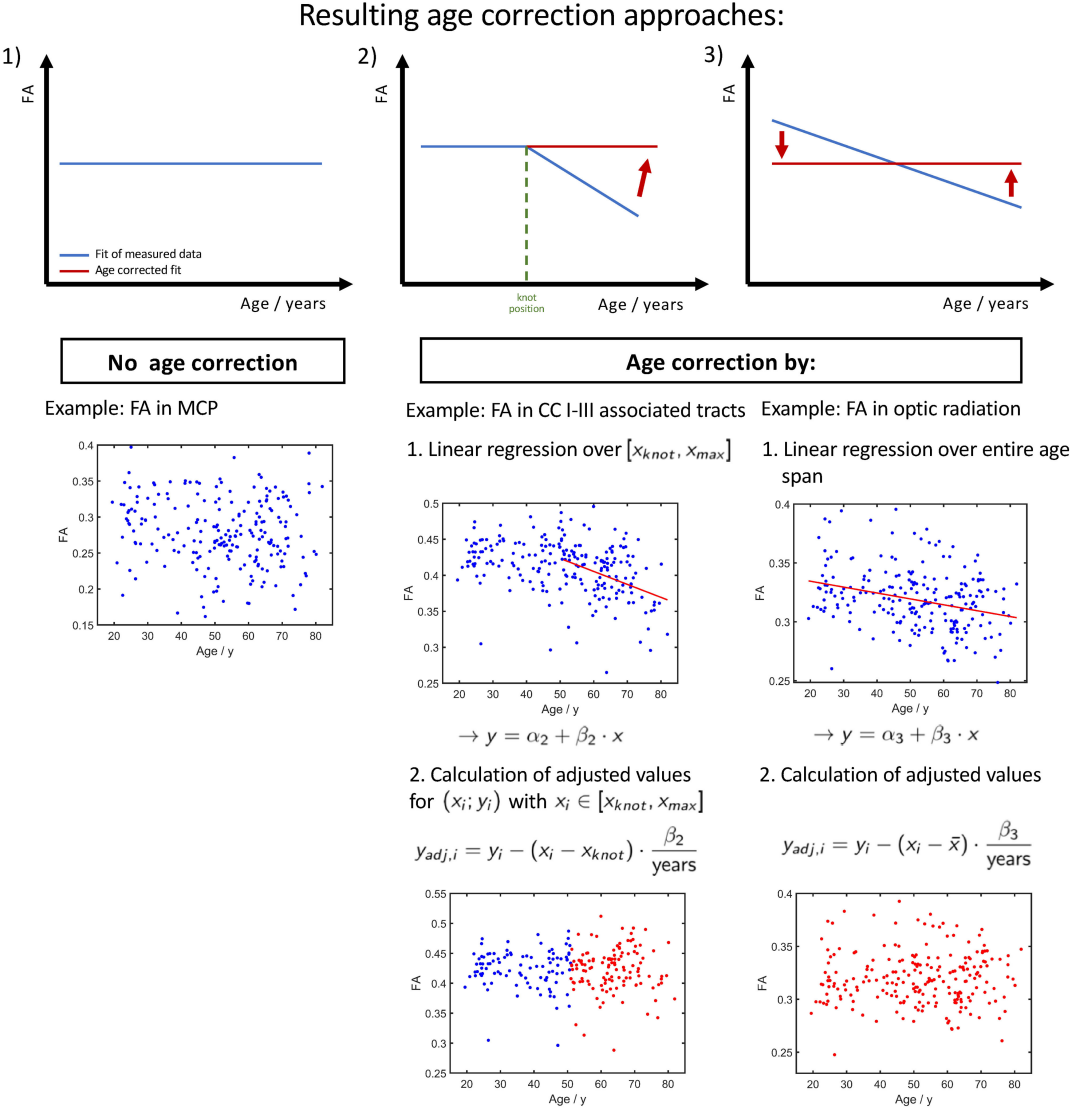
Resulting three different ways of age corrections of FA. (1) Tracts and age ranges where FA remains relatively stable. No correction for age necessary. (2) Age span encloses knot position x_knot_, i.e., a change of tendency in FA. The age correction is applied only on the time range of FA decrease. (3) Measured FA shows a decrease over the entire investigated age span, and the age correction can be applied to the entire age range.

Depending on the investigated tract system and the age range of the study cohort whose diffusion properties are to be age-corrected, we propose three different approaches, as shown in Figure 8. If the fit of measured data shows no or just a slight slope (Figure 8, constellation 1), there is no age correction necessary. Examples are the FA in the CST between 20 and 50 years of age or the MD in the cingulum between 20 and 50 years of age. Since the cerebellar microstructure seems to be unaffected by age with respect to DTI metrics, an age correction of the diffusion parameters is apparently not necessary in this area.

The second age correction approach (Figure 8, constellation 2) refers to tract systems and study group age ranges in which a change of alteration rate occurs from a plateau to a linear decrease/increase in diffusion properties; i.e., the interpolation with linear splines resulted in a lower $R_{adj}^{2}$ (see Table 2). When deciding whether a cohort's age range is in a pure plateau phase or is already affected by an incipient decrease/increase, the SD of the knot position should also be taken into account (see Table 3). Since the stable age phase does not need to be corrected, only the values above the knot position *x*_*knot*_ should be age corrected with respect to age at which the knot position occurs using equation (2) with the regression coefficient β_2_ from the linear regression of the age interval from the knot position to maximum age. For example, one age range, to which this applies, in tracks associated with CC areas I and III is 45–80 years.

As the third age correction approach for elder cohorts in whom the diffusion properties show a linear association, the age correction should be based on linear regression with regression coefficient β_3_ with respect to the mean or median of the group age (Figure 8, constellation 3).

In general, it can be inferred from the location of the knots in linear spline interpolation that younger cohorts (i.e., with a maximum age of about 40 years) do not need to be age-corrected, whereas cohorts of exclusively aged individuals (i.e., a minimum age about 60 years) can be corrected based on a linear regression. In contrast, the age correction of cohorts in whose age ranges the alteration rate of diffusion properties does not remain constant needs to be considered in more detail.

**Figure 9:**
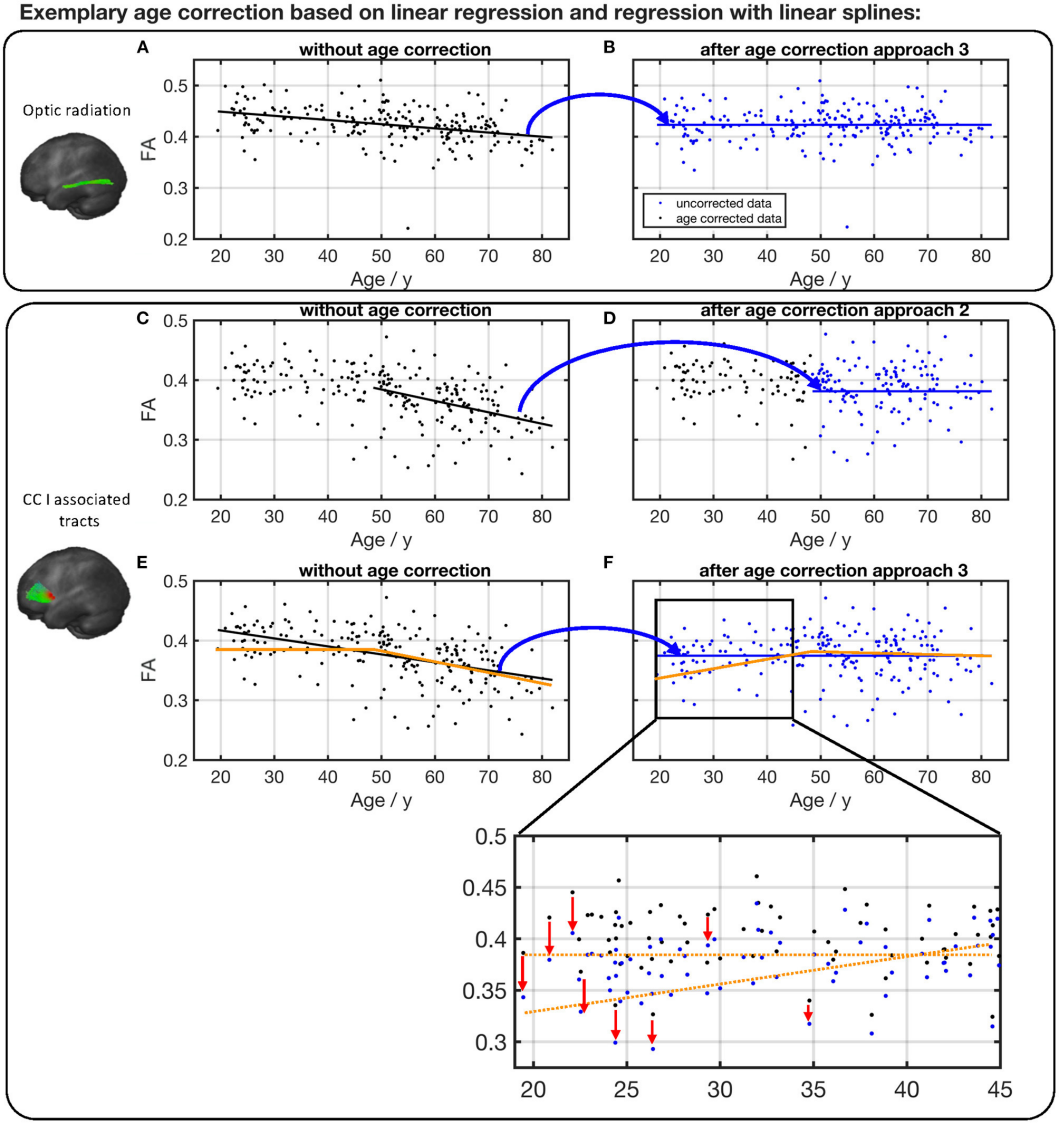
Comparison of different age correction approaches for the exemplary tract optic radiation **(A,B)** and CC area I-associated tracts **(C–F)**. **(A)** Measured FA values in optic radiation and linear regression line before age correction. **(B)** FA after age correction approach 3 (see Figure 8), based on linear regression over all participants' age span. Due to age correction, the regression of the corrected data shows a constant FA. **(C)** Measured FA values in CC I-associated tracts and linear regression line of ages higher than the estimated knot position. **(D)** FA after age correction approach 2 (see Figure 8). **(E)** Measured FA values and linear regression (black) over all participants' age span. The orange line serves as a guide to the eye and demonstrates the two-phase age-associated behavior. **(F)** FA after age correction approach 3. The red arrows in the zoom demonstrate on some examples the shift in FA values at the lower end of the age range due to an age correction based on a linear regression over the complete age range.

Specific examples (optic radiation vs. CC area I tracts) are shown in Figure 9. The FA in optic radiation shows a linear behavior throughout the life span. Therefore, the values here can be corrected based on a linear region (Equation 1) over the complete age span of the cohort (Figures 9A,B). The situation is different in the CC area I-associated tracts. Here, FA shows a biphasic behavior, and values below the knot position do not need to be corrected. For the FA values of participant ages above the knot position, Equation (2) would be applied for correction (Figures 9C,D). In contrast, an age correction in this tract system based on Equation (1) would lead to a clear shift of the values at the lower end of the age range (Figures 9E,F).

Our study was not without limitations. Since recruitment may be considered to be a random process, the age of our cohort distribution does not perfectly follow a uniform distribution. The fact that the number of subjects well advanced in years is reduced could possibly increase the variance of those at maximum age. In general, the variance of diffusion measures is quite high, so in some cases, a linear regression and a segmented regression describe the behavior of the properties similarly well; i.e., the adjusted coefficients of determination of both fits are very close to each other. Since our study cohort contains only adults, we are unable to draw conclusions about the transition from the increase in FA, or the decrease in MD, in childhood to the stable phase during adulthood. Therefore, a peak FA and minimum MD in early adulthood (Kochunov et al., 2012; Lebel et al., 2012) could be underestimated in our study. A further limitation is that data were recorded with different protocols and different scanners. However, DTI metrics' results do not show any bias after scanner and protocol homogenization so that the applicability to different MRI protocols might even be regarded as a strength of the study. In the application to other studies, it has to be noted that eddy current distortions were checked for, but no other corrections for susceptibility distortions in the data, e.g., top-up, were performed so that the results fail to account for the interaction of the susceptibility field with head movement, which can potentially lead to increased errors in the analysis of the diffusion MRI data (Graham et al., 2017).

In summary, this study contributes to the always emerging question on how age correction in DTI group studies has to be performed and provides concrete approaches for age correction based on FT techniques. When corrections for age in diffusion metrics are be carried out, spatial differences of the areas under investigation as well as the age range of the study cohort have to be taken into account.

## Data Availability Statement

The original contributions presented in the study are included in the article/Supplementary Material, further inquiries can be directed to the corresponding author/s.

## Ethics Statement

The studies involving human participants were reviewed and approved by Ethics Committee of the University of Ulm. The patients/participants provided their written informed consent to participate in this study.

## Author Contributions

AB: analysis and interpretation of the data and drafting the manuscript. JK: conceptualization of the study, interpretation of the data, and revising the manuscript for intellectual content. H-PM: design and conceptualization of the study, analysis and interpretation of the data, and revising the manuscript for intellectual content. All authors contributed to the article and approved the submitted version.

## Conflict of Interest

The authors declare that the research was conducted in the absence of any commercial or financial relationships that could be construed as a potential conflict of interest.
