# Supplementary material for: Age-Related Alterations in DTI Metrics in the Human Brain—Consequences for Age Correction
Source: Front Aging Neurosci. 2021 Jun 15;13:682109. doi: 10.3389/fnagi.2021.682109 (PMC8239142; doi:10.3389/fnagi.2021.682109)
Supplement: Supplementary file 1 [file Table_1.DOCX]

Supplementary Material

| Age range | 20-40 years | | | 40-60 years | | | 60-80 years | | |
| --- | --- | --- | --- | --- | --- | --- | --- | --- | --- |
| Age correction approach | 1 | 2 | 3 | 1 | 2 | 3 | 1 | 2 | 3 |
| CST |  |  | x |  |  | x | x |  |  |
| Frontooccipital tract | x |  |  |  | x |  |  | x |  |
| Fasciculus uncinatus |  |  | x |  |  | x | x |  |  |
| Optic radiation |  |  | x |  |  | x |  |  | x |
| SLF |  |  | x |  |  | x | x |  |  |
| ILF | x |  |  |  |  | x |  |  | x |
| Cingulum | x |  |  |  | x |  |  |  | x |
| SCP | x |  |  | x |  |  | x |  |  |
| MCP | x |  |  | x |  |  | x |  |  |
| Corticostriatal path | x |  |  |  |  | x |  |  | x |
| Corticopontine tract | x |  |  | x |  |  |  |  | x |
| Corticorubral tract | x |  |  |  |  | x |  |  | x |
| Perforant path | x |  |  |  | x |  |  |  | x |
| Temporal lobe to hypothalamus |  |  | x |  | x | x |  |  |  |
| AIC |  |  | x |  |  | x |  |  | x |
| PIC |  |  | x |  | x | x |  |  |  |
| CC I associated tracts | x |  |  |  | x |  |  |  | x |
| CC II associated tracts | x |  |  |  | x |  |  |  | x |
| CC III associated tracts | x |  |  |  | x |  |  |  | x |
| CC IV associated tracts | x |  |  |  |  | x |  |  | x |
| CC V associated tracts | x |  |  |  | x |  |  | x |  |

**Table 1** Exemplary overview of which age correction approach should be applied at which age range (here: 20-40y, 40-60y and 60-80y) of study cohorts. Age correction approach 1 stands for no age correction. Approach 2 means just a part of the cohort should be corrected for age. Approach 3 refers to age correction based on linear regression over the entire age range of the study cohort.
